# Association between attempted suicide and academic performance indicators among middle and high school students in Mexico: results from a national survey

**DOI:** 10.1186/s13034-018-0215-6

**Published:** 2018-01-24

**Authors:** Ricardo Orozco, Corina Benjet, Guilherme Borges, María Fátima Moneta Arce, Diana Fregoso Ito, Clara Fleiz, Jorge Ameth Villatoro

**Affiliations:** 1 Department of Epidemiology and Psychosocial Research, National Institute of Psychiatry (Mexico), Calzada Mexico-Xochimilco No. 101, Col. San Lorenzo Huipulco, 14370 Mexico City, Mexico; 2 General Office of Psychiatric Services, Ministry of Health (Mexico), Av. Paseo de la Reforma No. 450 Piso 1, Col. Juárez, 06600 Mexico City, Mexico

**Keywords:** Suicide, Attempted, Academic performance, Epidemiology

## Abstract

**Background:**

Students’ mental health is associated to academic performance. In high income countries, higher students’ grades are related to lower odds of suicidal behaviors, but studies on other indicators of academic performance are more limited, specially in middle income countries.

**Methods:**

Data from 28,519 middle and high school students selected with multistage clustered sampling in the Mexican National Survey of Student’s Drug Use. Using a self-administered questionnaire, lifetime suicidal attempt and four indicators of academic performance were assessed: age inconsistency with grade level, not being a student in the last year, perceived academic performance and number of failed courses. Multiple logistic regression models were used to control for sociodemographic and school characteristics.

**Results:**

The lifetime prevalence of attempted suicide was 3.0% for middle school students and 4.2% for high school students. Among middle school students, statistically adjusted significant associations of suicide attempt with academic performance indicators were: not being a student the year before, worse self-perceived performance and a higher number of failed courses; among high school students, predictors were failed courses and self-perceived academic performance, with ORs of 1.65 and 1.96 for the categories of good and fair/poor respectively, compared to those who reported very good performance.

**Conclusion:**

Self-perceived academic performance was the main indicator for suicide in both school levels. Suicide prevention efforts in Mexico’s schools should include asking students about the perception they have about their own academic performance.

## Background

According to the Global Burden of Disease Study, suicide is the leading cause of death for children and adolescents from 10 to 19 years of age living in developing countries. Among the 10–14 year old population, suicide has gone from the 14th place in 1990 to the 10th in 2013, increasing 17%; among young people aged 15–19, suicide has remained the second cause of death, but has increased by 18% [1]. In Mexico, completed suicide rates have been constant and steadily increasing, being of particular concern among the young population, increasing rapidly in the group of 15–29 year olds [2]. Population surveys have estimated that one in every 100 Mexican students made a suicide attempt in the previous year [3].

Peer relationships, teachers and families have a significant impact on academic performance, as well as on mental health and suicidal behaviors during school years [4]. Previous studies [5] show that mental health is associated with academic performance, as the latter is an important source for the development of identity, the development of social relationships between peers, the improvement of skills such as critical thinking and problem solving, and because it contributes to better opportunities for the future.

Cohort studies with vital statistics in Sweden have estimated that the odds of a serious suicide attempt in students decreased 60% for each point increase in its grading system (range 1–5) [5]. Some cross-sectional studies have reported an association between low grades and statistically significant increases of twice the odds of suicidal ideation and suicidal plan, but not with suicide attempts [6]. Other studies have established a fivefold increased likelihood of a suicide attempt among students with low perceived academic performance compared to those who rated their achievement as above average [7].

Epidemiologic studies in Mexican students have a long tradition [8, 9], mainly through local surveys of students living in Mexico City, but also through national ones. A study in 2000 found that, among 802 females students in Mexico City who had attempted suicide, 5% did it because of poor academic performance [10]. A national study in 2007, which included public schools only (n = 12,424), estimated that the prevalence of attempted suicide among high school students who reported low academic recognition was 12 and 8% among those with high academic recognition with an adjusted Odds Ratio (OR) of 1.04 (0.84–1.30) [11]. However, academic recognition is only one indicator of academic performance, and studies are needed which focus on identifying other indicators which may be associated with suicidal behaviors, to inform how to better implement effective suicide prevention programs in schools. Such policies are needed since the goal of member States of the World Health Organization (WHO)—including developing nations—is to reduce suicide rates by 10% by 2020 [12].

The purpose of this paper is to describe the national prevalence of suicide attempts among Mexican students, their distribution through different population groups and to estimate the magnitude of the association between suicide attempts and four indicators of academic performance, independent of other sociodemographic variables. We analyze a recent, large national epidemiologic survey (n = 28,519) that covered both public and private schools in rural and urban areas. Our hypothesis is that students with worse indicators of academic performance have a higher prevalence of suicide attempts.

## Methods

### Population and sample

The National Survey of Student’s Drug Use (Encuesta Nacional de Consumo de Drogas en Estudiantes—ENCODE) is a national survey of urban and rural schools in Mexico, selected using stratified clustered random sampling. In 2014, ENCODE’s target population included middle (12–14 years of age) and high school students (15–17 years of age) from all the country. Strata were formed by school level (middle and high school), state (all 32 Mexican States) and nine cities (Acapulco, Tijuana and Ciudad Juarez, among others) that were of special interest. The sample frame was formed by public and private schools: 34,733 middle and 12,841 high schools, excluding those from towns with more than 60% indigenous population and some specialized schools (e.g. for migrants).

In every school, classrooms were randomly selected by systematic sampling with random start according to the average number of students per class in each level [13]. All students in the classroom answered the questionnaire. It was not possible to conduct the survey in 61 of the selected classrooms due to safety issues in several municipalities. The response rate was 89.4%.

Data were weighted based on selection probabilities and subsequently adjusted for distribution of students by grade within each stratum. The ENCODE sample consists of 114,364 students (57,402 from middle school and 56,962 from high school). Academic performance indicators were asked only to a 25% random subsample. Hence, the sample size used for all analyses was n = 28,519; 14,435 middle school and 14,084 high school students.

### Instruments

Data were obtained from a self-administered questionnaire which was standardized, validated and administered in previous surveys [14]. The questionnaire consists of a main section, answered by all participants (sociodemographic information, substance use, antisocial behavior, social environment, among others) and four extra questionnaires that were applied only to a random sample of a quarter of the students each. For this paper we analyzed the sections of sociodemographic characteristics, suicide attempts and academic performance, which were included in one of the random samples.

### Main measurements

#### Lifetime suicide attempt

Based on González-Forteza’s “Parasuicide Indicator Data Sheet” (PIDS) [15], students where coded as suicide attempters if they: (1) responded positively to the question: “Have you ever injured, cut, poisoned or harmed yourself in order to take your life?” and, (2) gave valid answers to follow-up questions about: age at the only (or last) attempt, the motive, method and indicators of seriousness [10] and, (3) confirmed that they tried to “[…]hurt yourself on purpose in order to take your life?”.

#### Academic performance

For the present study, we created four variables of academic performance which have also been used in previous research [16–18]: (1) *age inconsistency with grade level*, students who reported being 2 or more years older than the expected age and year level that they were studying during the survey; (2) *Not being a student in the last year*, students who reported that did not attend school the previous year; (3) *Perceived academic performance*, which was measured with the question: “In general, how do you consider your academic performance in school?” with four possible answers: very good, good, regular and bad; (4) *Number of failed courses*, divided into four categories: none, one, two, and three or more.

### Covariates

#### Sociodemographic characteristics

The sociodemographic characteristics considered included sex, age, having a job most of the previous year and if it was full or part time, speaking an indigenous language, size of the locality where the student has lived most of his/her life (big, medium or small city, small town/rural community), family constellation (living with: both parents, both parents but one is a surrogate, single mother (or surrogate), single father (or surrogate) or others), mother’s (or surrogate’s) education level and father’s (or surrogate's) education level.

#### School characteristics

The school characteristics considered were the school shift (morning, afternoon and other, such as full time or extra time) and school grade (in Mexico, 7th, 8th and 9th grades are equivalents to the three grades of middle school and 10th, 11th and 12th to the three grades of high school, even though in México middle and high school are divided separately into 3 grades each).

### Statistical analyses

The bivariate analysis consisted of frequencies and percentages for contingency tables with categorical variables. Comparisons between categories were conducted using the Chi square Pearson statistic, corrected for by the survey design. Statistical significance was assessed with the *p* value less than 0.05. Multiple logistic regression models were performed, with attempted suicide as the dependent variable, each academic performance as the main independent variable and sociodemographic characteristics and school characteristics as covariates. In the final models for either middle or high school, only variables with p < 0.20 in the bivariate models were entered as covariates. Further pairwise comparisons for significant variables with three or more categories were performed using Stata’s *test* command.

All statistical analyses were stratified by school level, in order to estimate associations for students in middle and high school separately. Data were analyzed in Stata version 13.1 [19] using the module for analysis of complex surveys *svy*, which corrects standard errors through the Taylor series method [20], based on the sample design, weighting and clustering of observations.

## Results

The analysis of the sociodemographic composition of Mexican students shows slightly more women in high school (51.2%) than in middle school (49.5%) (Table 1). Just over 5% of middle school students were 15 years or older and no young people under 14 attended high school. Approximately two out of ten students worked either full or part time during the previous year, and having a part-time job was reported more frequently by high school students than middle school students (p < 0.001). Less high school students spoke an indigenous language and lived most of their lives in small towns or rural areas as compared to their middle school counterparts. 75% of middle school students lived with both parents, decreasing to 72% for high school students. Level of education both for father and mother (or their surrogates) was higher in high school students than in middle school ones. In terms of school characteristics, three quarters of middle schoolers attended at morning shift (78%), as well as 58% of high school students; at both levels the highest proportion of students was concentrated in the 7th and 10th year (41.1% for middle school students and 42.2% for high school students).

**Table 1:** Sociodemographic and school characteristics of Mexican public and private school students. Mexico, 2014

	Level						*X* ^2^	df	p value
	Middle school		High school		Total				
	(n = 14,435)		(n = 14,084)		(n = 28,519)				
	n	%	n	%	n	%			
Sex							31.8	1	0.289
Male	7253	50.5	6990	48.8	14,243	49.9			
Female	7182	49.5	7094	51.2	14,276	50.1			
Age (years)							94,470	8	< 0.001
11	734	5.8	–	–	734	3.6			
12	4463	31.4	–	–	4463	19.4			
13	4874	31.9	–	–	4874	19.7			
14	3515	25.0	414	3.2	3929	16.6			
15	717	4.9	4527	29.5	5244	14.3			
16	93	0.7	4457	33.5	4550	13.2			
17	25	0.2	3292	23.6	3317	9.2			
18	13	0.1	918	6.2	931	2.4			
19–29	1	0.0	476	3.9	477	1.5			
Worked the year before							544.0	2	< 0.001
No	11,422	79.2	10,690	77.6	22,112	78.6			
Yes, part time job	1550	11.7	2266	15.7	3816	13.2			
Yes, full time job	1164	9.1	941	6.7	2105	8.2			
Speaks an indigenous language							401.0	1	0.001
No	13,231	94.2	13,329	96.8	26,560	95.2			
Yes	784	5.8	420	3.2	1204	4.8			
Size of locality							323.0	2	0.182
Big city	3650	24.5	3853	28.1	7503	25.9			
Medium/small city	6450	40.9	6555	42.0	13,005	41.3			
Small town or rural area	4155	34.6	3567	29.9	7722	32.8			
Family structure							140.8	4	0.006
Both parents	10,595	74.5	9954	72.5	20,549	73.8			
Both parents (one surrogate)	899	5.3	767	4.7	1666	5.1			
Mother (or surrogate)	2202	15.1	2477	17.2	4679	15.9			
Father (or surrogate)	325	2.2	328	2.2	653	2.2			
Other	414	2.8	558	3.4	972	3.0			
Mother’s (or surrogate's) education level							3059.6	5	< 0.001
Elementary or no education	3590	28.2	3096	22.1	6686	25.9			
Middle school	4523	31.3	4379	30.8	8902	31.1			
High school	2493	17.0	3442	24.5	5935	19.9			
University/college	1428	9.3	1718	12.5	3146	10.5			
Postgraduate studies	1021	6.2	1056	7.8	2077	6.8			
Other	1088	7.9	283	2.2	1371	5.7			
Father’s (or surrogate's) education level							3092.5	5	<0.001
Elementary or no education	3439	27.2	2919	20.7	6358	24.7			
Middle school	4170	29.7	3949	29.0	8119	29.5			
High school	2426	16.4	3274	23.8	5700	19.2			
University/college	1508	10.0	1937	14.2	3445	11.7			
Postgraduate studies	1115	6.6	1194	8.4	2309	7.3			
Other	1427	10.0	515	3.8	1942	7.6			
School shift							5271.1	2	< 0.001
Morning	11,038	77.7	8688	58.9	19,726	70.5			
Afternoon	3145	19.3	4156	30.8	7301	23.7			
Other	252	3.1	1240	10.2	1492	5.8			
School year^a^							453.9	2	0.437
First (7th, 10th)	5711	41.1	6542	42.2	12,253	41.5			
Second (8th, 11th)	5471	34.5	3826	29.1	9297	32.4			
Third (9th, 12th)	3253	24.3	3716	28.7	6969	26.0			

Missing Values: Worked last year (486); indigenous language speaker (755); place of residence (289); mother’s education (402); father’s education (646)

Percentages are weighted, frequencies are unweighted; p value adjusted due to the survey design

^a^7th, 8th and 9th grades as equivalents to the three grades of middle school and 10th, 11th and 12th for the three grades of high school. In México, middle and high school are divided into 3 grades each

The lifetime prevalence of attempted suicide was 3% for middle school students and 4.2% for high school students. In both middle and high school students, the prevalence of attempts in women (5.2 and 6.8%) was higher than in men (1.1 and 1.5%) with a statistically significant difference (Table 2). In relation to other variables, the highest prevalence rates in middle school students were estimated among students who were enrolled in their second or third year. For the variables of academic performance, the only statistically significant difference was observed among middle school students, with a higher proportion of suicide attempts among those who rated their academic performance as fair or poor (3.8%) compared to those who perceived it as good (3.2%) or very good (1.8%), p = 0.011.

**Table 2:** Prevalence of attempted suicide by sociodemographic, school and academic performance variables

	Level											
	Middle school (n = 14,435)						High school (n = 14,084)					
	Sample	Attempts	%	*X* ^2^	df	p value	Sample	Attempts	%	*X* ^2^	df	p value
Sociodemographic and school												
Sex				803.0	1	< 0.001				963.0	1	< 0.001
Male	7198	75	1.1				6963	119	1.5			
Female	7136	389	5.2				7069	471	6.8			
School shift				16.9	2	0.406				3.9	2	0.805
Morning	10,963	336	3.1				8654	363	4.1			
Afternoon	3121	125	3.3				4141	172	4.5			
Other	250	3	1.5				1237	55	4.3			
School year^a^				220.3	2	< 0.001				31.0	2	0.169
First year (7th, 10th)	5667	100	1.8				6511	295	4.8			
Second year (8th, 11th)	5436	218	4.0				3819	145	3.8			
Third year (9th, 12th)	3231	146	3.9				3702	150	3.9			
Academic performance												
Two years older than expected for grade level				5.6	1	0.357				19.5	1	0.162
No	13,797	452	3.1				12,831	529	4.1			
Yes	537	12	2.3				1201	61	5.4			
Studying previous year				62.0	1	0.085				0.6	1	0.781
Yes	13,557	440	3.0				12,906	549	4.3			
No	478	19	6.2				891	37	4.0			
Perceived academic performance				94.9	2	0.011				49.9	2	0.052
Very good	2997	67	1.8				2082	64	2.8			
Good	6458	216	3.2				7489	308	4.3			
Fair or poor	4606	177	3.8				4305	213	4.7			
Number of failed courses				48.3	3	0.105				28.9	3	0.298
None	11,582	353	3.0				10,266	409	4.1			
1	952	41	3.3				1449	69	4.7			
2	615	30	3.2				838	43	5.9			
3 or more	631	30	5.4				992	55	4.2			

Missing Values: Attempted suicide (153; 101 middle school and 52 high school); studying the previous year (544); self-perceived school performance (512); failed courses (1126)

Percentages are weighted, frequencies are unweighted; p values adjusted due to the survey design

^a^ 7th, 8th and 9th grades as equivalents to the three grades of middle school and 10th, 11th and 12th for the three grades of high school. In México, middle and high school are divided into 3 grades each

Table 3 shows the estimates of adjusted ORs from multiple logistic regression models for middle school students. Significant predictors of suicide attempt related to academic performance were: not being a student the year before, worse self-perceived performance and having failed three or more courses. Compared to those who perceived themselves to have very good academic performance, those who reported only good performance had almost twice the odds of attempted suicide (OR = 1.86; 95% CI = 1.16–2.99), whereas those who reported having fair or poor performance had 2.35 times the odds (95% CI = 1.56–3.54), controlling for all other variables in the model (sex, age, shift, grade, etc.), further pairwise comparisons did not show significant differences in these two last estimates (p = 0.25). Regarding the number of failed courses, the only statistically significant association was observed between those who reported three or more failed courses compared with those with none, with an OR = 2.41 (95% CI = 1.26–4.60).

**Table 3:** Association between four school performance indicators and school sociodemographic variables in middle school students

	Two years older than expected for grade level			Studying last year			Perceived academic performance			Number of failed courses		
	(a) No			a) Yes			a) Very good			a) None		
	(b) Yes			b) No			b) Good			b) 1		
							c) Fair or poor			c) 2		
										d) 3 or more		

	(n = 13,624)			(n = 13,403)			(n = 13,380)			(n = 13,123)		
	OR	95% CI	Sig.	OR	95% CI	Sig.	OR	95% CI	Sig.	OR	95% CI	Sig.
School performance variable												
(a)	1.00	–		1.00	–		1.00	–		1.00	–	
(b)	0.83	(0.31–2.21)		2.75	(1.17–6.50)	*	1.86	(1.16–2.99)	*	1.18	(0.74–1.88)	
(c)	–	–		–	–		2.35	(1.56–3.54)	***	1.12	(0.63–1.98)	
(d)	–	–		–	–		–	–		2.41	(1.26–4.60)	**
Sex												
Male	1.00	–		1.00	–		1.00	–		1.00	–	
Female	5.79	(4.15–8.07)	***	5.74	(4.13–7.98)	***	6.45	(4.56–9.11)	***	6.05	(4.32–8.47)	***
Age (continuous)	1.02	(0.75–1.39)		0.98	(0.78–1.23)		0.98	(0.77–1.23)		0.96	(0.76–1.22)	
Worked last year												
No	1.00	–		1.00	–		1.00	–		1.00	–	
Yes, part-time	2.49	(1.51–4.10)	***	2.34	(1.48–3.71)	***	2.41	(1.43–4.06)	***	2.50	(1.51–4.14)	***
Yes, full-time	1.34	(0.71–2.51)		1.35	(0.71–2.57)		1.37	(0.72–2.59)		1.32	(0.69–2.52)	
Size of locality												
Big city	1.00	–		1.00	–		1.00	–		1.00	–	
City	1.01	(0.72–1.42)		1.01	(0.72–1.42)		1.01	(0.71–1.42)		1.00	(0.71–1.41)	
Small town or hamlet	0.80	(0.50–1.29)		0.78	(0.48–1.26)		0.82	(0.51–1.32)		0.79	(0.48–1.28)	
Family constellation												
Both parents	1.00	–		1.00	–		1.00	–		1.00	–	
Both parents (one surrogate)	1.37	(0.90–2.09)		1.37	(0.90–2.08)		1.32	(0.86–2.03)		1.33	(0.87–2.03)	
Mother (or surrogate)	1.12	(0.75–1.68)		1.09	(0.74–1.61)		1.11	(0.74–1.67)		1.09	(0.73–1.63)	
Father (or surrogate)	2.42	(1.06–5.48)	*	2.44	(1.07–5.58)	*	2.50	(1.08–5.77)	*	2.50	(1.08–5.79)	*
Other	1.88	(1.02–3.45)	*	1.89	(1.02–3.48)	*	1.93	(1.04–3.56)	*	1.83	(0.95–3.51)	
Mother’s (or surrogate’s) education level												
Elementary or no education	1.00	–		1.00	–		1.00	–		1.00	–	
Middle school	1.25	(0.84–1.86)		1.24	(0.82–1.86)		1.25	(0.84–1.85)		1.25	(0.84–1.86)	
High school	1.37	(0.89–2.10)		1.38	(0.90–2.12)		1.41	(0.91–2.17)		1.35	(0.88–2.09)	
University/college	1.57	(0.97–2.54)		1.57	(0.97–2.54)		1.70	(1.03–2.79)	*	1.53	(0.94–2.49)	
Postgraduate studies	1.50	(0.78–2.87)		1.53	(0.80–2.94)		1.62	(0.84–3.12)		1.52	(0.79–2.91)	
Other	0.61	(0.33–1.13)		0.60	(0.32–1.11)		0.58	(0.30–1.10)		0.55	(0.29–1.06)	
School year^a^												
First year (7th)	1.00	–		1.00	–		1.00	–		1.00	–	
Second year (8th)	2.15	(1.25–3.70)	**	2.42	(1.47–3.96)	***	2.19	(1.36–3.52)	**	2.30	(1.41–3.74)	***
Third year (9th)	2.03	(0.96–4.28)		2.38	(1.30–4.37)	**	2.04	(1.10–3.78)	*	2.29	(1.23–4.28)	**

*OR* Odds Ratio, *95% CI* 95% Confidence Interval

* p  < 0.05; ** p < 0.01; *** p < 0.001

^a^ 7th, 8th and 9th grades as equivalents to the three grades of middle school in México

Adjusted estimates for high school students are shown in Table 4. After controlling for sociodemographic and school characteristics variables, the statistically significant predictors were having failed two courses compared to none (OR = 1.78; 95% CI = 1.10–2.86) and self-perceived academic performance, with associations of 1.65 (95% CI = 1.08–2.52) and 1.96 (95% CI = 1.25–3.06) for the categories of good and fair/poor respectively, compared to those who reported very good performance. Again, further pairwise comparisons did not reveal significant differences in these two last estimates (p = 0.22).

**Table 4:** Association between the school performance indicators and school sociodemographic variables in high school students

	Two years older than expected for grade level			Studying last year			Perceived academic performance			Number of failed courses		
	(a) No			(a) Yes			(a) Very good			(a) None		
	(b) Yes			(b) No			(b) Good			(b) 1		
							(c) Fair or poor			(c) 2		
										(d) 3 or more		

	(n = 13,423)			(n = 13,219)			(n = 13,279)			(n = 12,970)		
	aOR	95% CI	Sig.	aOR	95% CI	Sig.	aOR	95% CI	Sig.	aOR	95% CI	Sig.
Academic performance variable												
(a)	1.00	–		1.00	–		1.00	–		1.00	–	
(b)	1.31	(0.64–2.67)		0.84	(0.49–1.44)		1.65	(1.08–2.52)	*	1.36	(0.93–2.00)	
(c)	–	–		–	–		1.96	(1.25–3.06)	**	1.78	(1.10–2.86)	*
(d)	–	–		–	–		–	–		1.40	(0.90–2.18)	
Sex												
Male	1.00	–		1.00	–		1.00	–		1.00	–	
Female	4.86	(3.59–6.58)	***	4.80	(3.54–6.49)	***	5.01	(3.67–6.84)	***	5.25	(3.84–7.19)	***
Age (continuous)	1.04	(0.87–1.24)		1.09	(0.96–1.23)		1.08	(0.97–1.21)		1.08	(0.96–1.21)	
Speaks an indigenous language												
No	1.00	–		1.00	–		1.00	–		1.00	–	
Yes	0.51	(0.24–1.06)		0.52	(0.25–1.09)		0.52	(0.25–1.07)		0.54	(0.26–1.12)	
Family constellation												
Both parents	1.00	–		1.00	–		1.00	–		1.00	–	
Both parents (one surrogate)	1.99	(1.25–3.15)	**	2.00	(1.26–3.18)	**	1.98	(1.24–3.15)	**	1.97	(1.23–3.16)	**
Mother (or surrogate)	1.82	(1.36–2.44)	***	1.84	(1.38–2.47)	***	1.81	(1.35–2.42)	***	1.87	(1.40–2.49)	***
Father (or surrogate)	1.39	(0.61–3.16)		1.44	(0.63–3.28)		1.36	(0.59–3.11)		1.37	(0.59–3.22)	
Other	1.52	(0.92–2.51)		1.47	(0.88–2.48)		1.49	(0.90–2.46)		1.51	(0.89–2.54)	
Father’s (or surrogate´s) education level												
Elementary or no education	1.00	–		1.00	–		1.00	–		1.00	–	
Middle school	1.30	(0.97–1.76)		1.32	(0.97–1.78)		1.30	(0.96–1.77)		1.34	(1.00–1.80)	
High school	0.94	(0.65–1.36)		0.93	(0.64–1.35)		0.96	(0.66–1.39)		0.98	(0.67–1.41)	
University/college	0.82	(0.53–1.29)		0.82	(0.53–1.28)		0.85	(0.54–1.32)		0.86	(0.55–1.35)	
Postgraduate studies	0.99	(0.63–1.56)		0.98	(0.62–1.55)		1.01	(0.63–1.62)		1.01	(0.64–1.60)	
Other	0.97	(0.54–1.75)		0.92	(0.51–1.67)		1.00	(0.56–1.80)		0.90	(0.49–1.63)	
School year^a^												
First year (10th)	1.00	–		1.00	–		1.00	–		1.00	–	
Second year (11th)	0.75	(0.53–1.05)		0.69	(0.50–0.95)	*	0.71	(0.52–0.96)	*	0.70	(0.51–0.95)	*
Third year (12th)	0.74	(0.47–1.18)		0.66	(0.44–1.00)		0.69	(0.47–1.01)		0.63	(0.42–0.95)	*

*aOR* Adjusted Odds Ratio,* 95% CI* 95% Confidence Interval

* p < 0.05; ** p < 0.01; *** p < 0.001

^a^10th, 11th and 12th for the three grades of high school in México

## Discussion

In Mexico, prior estimates of the lifetime prevalence of attempted suicide among students vary from 1.4% of middle school students and 2% of high school students, [21], up to 9% in high school students [11]. In this paper we estimated a prevalence of 3% in students from middle school and 4.2% for those attending high school. While other national studies have used a single question to identify suicide attempts, in our study we used a battery of questions that increased the instrument’s sensitivity to detect young people with a genuine suicide attempt. The results are very similar to those reported by Mexican adolescents in the general population (3.1%) obtained through other instruments like the WHO Composite International Diagnostic Interview [22].

On the other hand, while the prevalence of attempted suicide increases with school year in students attending middle school (probably due to stress related to adjustments to adolescence), as it goes from 1.8 in the 7th year to practically 4 in 8th and 9th grade, among the high school population the prevalence decreased. Because the data comes from a survey, it is possible that school dropout plays a role in the prevalence of attempts, especially in the high school level: in Mexico, only 57% of the population between 15 and 18 attends school [23], with a dropout rate of 15.9% [24]. The latter could be explained because young people with major mental health problems, including suicide, are most likely to leave school at this level, thus, the prevalence diminishes through this selection effect.

Of the four indicators of academic performance we studied, only perceived academic performance was associated to suicide attempt in middle school students in bivariate analysis. After adjustment for potential confounders, self-perceived academic performance was identified as a risk factor for suicide attempt, suggesting a dose–response for both school levels. This is consistent with other findings reported in both longitudinal and cross-sectional studies [5, 7, 25]. In middle school students, those who did not attend school the previous year had higher odds of suicidal attempt. Regarding the number of failed courses, we found a significantly higher prevalence among middle school students who failed three or more, and in high school students among those with two. Therefore, it would be appropriate to identify students that have a higher number of failed courses in order to screen them for suicidal behaviors. Since the number of failed courses was self-reported, caution must be exerted in the interpretation of this results, since students might conceal that they failed a course or, quite the contrary, to over-report them by interpreting the question as not doing well.

The fourth indicator that we studied, being 2 years older for their school grade, was not associated with suicidal attempt among this population. Nevertheless, this indicator could be related with other suicidal behaviors such as ideation or suicidal plan, which at the same time are precursors to more serious behaviors [17, 26, 27]. Given that our subjective indicator (perceived academic performance) was consistently more associated with attempts than the objectives ones (like age inconsistency with grade level), it is possible that cognitive distortions resulting from depressive states change student’s perceptions of academic achievement, being this a consequence of poor mental health instead of a real decline of academic performance. Future investigations should research this area.

In middle school students three sociodemographic risk factors were identified in the self-perceived academic performance models: sex, having worked part time and the type of family structure. Women are generally at greater risk of suicidal ideation plan and attempt [28] and in our study being female was the largest predictor of suicide attempt. It is noteworthy that students who are studying and working part-time rather than full-time are at the greatest risk, yet this association has been documented elsewhere [29]. It is likely that families’ financial stress is the main driving force that makes middle schoolers to look for a job, putting them at increased burden. Our results suggest that prevention programs in middle schools may screen students for suicidal behaviors, among those who share this burden, or who have left school for a year and came back.

Because this is a cross-sectional study, its main limitation is the impossibility to estimate the incidence of suicide attempts in the student population since the first follow-up year. It is likely that students with mental health problems and suicide attempts abandon school [30], so it is very important to identify and treat currently enrolled students who have these behaviors, since only half of adolescents who reported suicide attempts received mental health care once in a lifetime [22]. Furthermore, with this design we are not able to determine the direction of the association (timing) between our academic performance indicators and suicidal attempts, and it is possible that some mental health problems, such as depression, are risk factors for low academic performance [31].

Another limitation of the study was measuring suicide attempts: despite the use of the PIDS, a scale proved and used throughout the years in Mexico, in this study we incorporate the criterion that students confirm their suicidal action, with the intention of increasing the sensitivity of the measurement of an actual attempt and not only self-harming behavior (deliberate self-harm) [15]. The effect of this criteria could (providing that this effect it is non-differential for dichotomous variables) underestimate the extent of association measures (i.e. OR) [32] so the magnitude of the relationship of suicide attempts with academic performance variables could be even higher than estimated.

Finally, this work does not take into account the role that psychiatric disorders have on suicide attempts, since they are one of its main risk factors. Studies in Mexico [33] indicate that young people with depression have a 16-fold greater risk of suicidal ideas and 5 times higher for suicide attempts compared with those without. Also, because the questionnaire was divided and applied in four different sub-samples, with the sections on academic performance and depression (which also included suicide thoughts) being applied separately, it was not possible to include any of these last measures in the analysis.

## Conclusions

Our results show that suicide prevention efforts in México’s schools may include assessing adolescents’ perception about their own academic performance. This recommendation could be implemented through “gatekeepers” such as teachers and school personnel, who can be trained in suicide prevention and in identifying people at risk in order to direct them to an evaluation and appropriate treatment. Moreover, suicide prevention efforts in the public education system should consider comprehensive interventions at the individual, selective and universal levels, as recommended by the WHO [34] with support from other branches of the government, such as the health and public security sectors, in order to consolidate a national suicide prevention program, with the intention to cover all the way from the adequate registration of suicidal behavior to the adequate reference for treatment of students with suicide attempts.

## Abbreviations

ENCODE: Encuesta Nacional de Consumo de Drogas en Estudiantes
WHO: World Health Organization
PIDS: Parasuicide Indicator Data Sheet
OR: odds ratio
CI: confidence interval
